# Acupuncture and Immunity

**DOI:** 10.1155/2015/260620

**Published:** 2015-08-05

**Authors:** Fengxia Liang, Edwin L. Cooper, Hua Wang, Xianghong Jing, Juan G. Quispe-Cabanillas, Tetsuya Kondo

**Affiliations:** ^1^Acupuncture and Moxibustion Institute, College of Acupuncture & Moxibustion, Hubei University of Chinese Medicine and Hubei Provincial Collaborative Innovation Center of Preventive Treatment by Acupuncture and Moxibustion, Wuhan 430061, China; ^2^Laboratory of Comparative Neuroimmunology, Department of Neurobiology, David Geffen School of Medicine at UCLA, University of California, Los Angeles, Los Angeles, CA 90095-1763, USA; ^3^Institute of Acupuncture and Moxibustion, China Academy of Chinese Medical Sciences, Beijing 110007, China; ^4^Neuroimmunology Unit, Department of Genetics, Evolution and Bioagents, University of Campinas, 13081-970 Campinas, SP, Brazil; ^5^Kansai University of Health Sciences, 2-11-1 Wakaba, Kumatori-cho, Sennan-gun 590-0482, Japan

As a traditional therapy applied for thousands of years, acupuncture has recently been attracting more and more investigators throughout the world. In the theory of traditional Chinese medicine, it is proposed that acupuncture can strengthen the human body to resist diseases by puncturing needles at certain points. The characteristic that acupuncture enhances resistance is closely related with the immune system, which functions in defense, homeostasis, and surveillance. More and more research has revealed that acupuncture can regulate immunity, for example, to enhance anticancer and antistress immune function and exert anti-inflammation effects. This may be the basis of acupuncture in preventing and treating later diseases. This special issue was developed to stimulate the continuing efforts in promoting the research on acupuncture and immunity.

The acupuncture point ST36 (Zusanli) is widely applied in immune-related diseases. In “Electroacupuncture at Bilateral Zusanli Points (ST36) Protects Intestinal Mucosal Immune Barrier in Sepsis” M. Zhu et al. reported that EA preconditioning at ST36 obviously ameliorated CLP-induced intestinal injury and high permeability and exerted protective effects on intestinal mucosal immune barrier by increasing the concentration of sIgA and the percentage of CD3+, *γ*/*δ*, and CD4+ T cells and the ratio of CD4+/CD8+ T cells, which eventually decreased the mortality of sepsis.

“Moxibustion and Acupuncture Ameliorate Crohn's Disease by Regulating the Balance between Th17 and Treg Cells in the Intestinal Mucosa” by C. Zhao et al. provided evidence from a randomized controlled clinical trial that moxibustion and acupuncture regulated the ratio of Th17 and Treg cells in the intestinal mucosa of CD patients and restored the balance between these immune cell subsets, providing the basis for clinical application of treatment for CD.

“Herb-Partitioned Moxibustion Regulates the TLR2/NF-*κ*B Signaling Pathway in a Rat Model of Ulcerative Colitis” by X. Wang et al. assessed the regulation of the TLR2/NF-*κ*B signaling pathway by herb-partitioned moxibustion in the intestinal mucosa of rats with ulcerative colitis (UC). It was reported that herb-partitioned moxibustion modulated the excessive local immune response by inhibiting TLR2 signaling, thereby promoting the repair of damaged colonic mucosa.

“Mediators, Receptors, and Signalling Pathways in the Anti-Inflammatory and Antihyperalgesic Effects of Acupuncture” by J. L. McDonald et al. reviewed both demonstrated and proposed anti-inflammatory effects of acupuncture and analyzed the complex crosstalk between receptors during inflammation to elucidate the mediators and signaling pathways activated by acupuncture. Researches with new advances which demonstrated that acupuncture activated a novel cholinergic anti-inflammatory pathway and chemokine-mediated proliferation of opioid-containing macrophages in inflamed tissues were also included.

“Immunoregulation on Mice of Low Immunity and Effects on Five Kinds of Human Cancer Cells of* Panax japonicus* Polysaccharide” by Z. Jie et al. investigated the immunoregulative effects of* Panax japonicus* polysaccharide (PJPS) on mice of low immunity. The results indicated that PJPS significantly improved the immune function of mice processed by cyclophosphamide and PJPS did not work on the five kinds of human cancer cells.

In summary, this issue provides different evidence presented by diverse authors covering several topics related to advances in acupuncture for inflammation or immune diseases. As inflammation is the coherent pathophysiologic progress in many kinds of diseases, immune system and response of the human body are influenced in diseases such as cancer; the anti-inflammation effect of acupuncture may be a very important underlying mechanism of acupuncture in treating diseases. Moreover, TCM focuses on the theory that prevention before the onset of the diseases or intervening in an early stage of diseases is much better than treating after the onset. Preconditioning by acupuncture which means stimulation with acupuncture before the onset of diseases has been widely applied from ancient time to present clinical practice, proposing a potential field in preventing diseases. Acupoints selection, combination of the acupoints, or different methods of stimulation of acupuncture may result in quite different effects on immune system. Further research is urgently essential to elucidate the relation between acupuncture and immune responses and what kind of stimulation might induce the best effect.

Of course, the selected topics and papers are not a comprehensive representation of the area of this special issue. Nonetheless, they represent many-faceted evidence that we have the pleasure of sharing with the readers.

